# Subclinical Myocardial Dysfunction in Pediatric and Adolescent Celiac Disease Patients: A Systematic Review and Meta-Analysis

**DOI:** 10.3390/children12040441

**Published:** 2025-03-30

**Authors:** Elpida Emmanouilidou-Fotoulaki, Maria Kavga, Michail Delis, Evangelia Farmaki, Charalampos Agakidis, Kyriaki Papadopoulou-Legbelou

**Affiliations:** 1 1st Department of Pediatrics, School of Medicine, Aristotle University of Thessaloniki, 54642 Thessaloniki, Greece; farmakg@auth.gr (E.F.); cagakidis@auth.gr (C.A.); 2 3rd Department of Pediatrics, School of Medicine, Aristotle University of Thessaloniki, 54642 Thessaloniki, Greece; mkavga@auth.gr; 3 1st Department of Obstetrics and Gynecology, School of Medicine, Aristotle University of Thessaloniki, 56403 Thessaloniki, Greece; mdelisa@auth.gr; 4 4th Department of Pediatrics, School of Medicine, Aristotle University of Thessaloniki, 56403 Thessaloniki, Greece; kpapadopoulou@auth.gr

**Keywords:** celiac disease, myocardial dysfunction, children, systematic review, meta-analysis

## Abstract

**Background/Objectives:** This is the first systematic review and meta-analysis investigating early myocardial dysfunction in children/adolescents with celiac disease and the effect of a gluten-free diet by comparing early echocardiographic markers between patients and healthy individuals and between compliant and non-compliant celiac disease patients (based on serum antibody titers). **Methods:** A systematic literature search was conducted across major electronic databases, with data collection extending up to 3 March 2024. **Results:** In total, 15 studies with 916 children/adolescent patients with celiac disease and 569 healthy individuals were included. Our results showed a trend toward reduced myocardial function in all echocardiographic parameters (conventional and advanced), with statistical significance in fractional shortening and the myocardial performance index. However, these parameters did not differ significantly after adherence to a gluten-free diet. **Conclusions:** Therefore, we recommend that an examination of the cardiovascular system should be incorporated into the routine investigations of children with celiac disease in order to detect early subclinical myocardial dysfunction based on echocardiography. Although the results of our meta-analysis indicate that the myocardial performance index may serve as a useful, non-invasive marker for assessing myocardial function in children and adolescents with celiac disease, further research is needed in order to confirm its reliability and clinical applicability in this population. The improvement of echocardiographic parameters after long-term compliance to a gluten-free diet is yet to be evaluated.

## 1. Introduction

Celiac disease is a chronic, multi-organ, autoimmune, small-intestinal enteropathy precipitated by gluten exposure in genetically predisposed individuals [1]. Incidence rates of celiac disease are rising over time [2], and global prevalence estimates are higher in children compared to adults (0.9% vs. 0.5%) [3]. Although the classic clinical manifestation is malabsorption syndrome, over the last decades, atypical phenotypes with extraintestinal symptoms or even asymptomatic disease have become more prevalent [4,5]. Moreover, even though celiac disease has been recognized as a systemic disease, and system-specific consequences are well described in the literature [6], scientific evidence on cardiac involvement remains inconsistent and sparse.

The most recent cardiovascular consequences that have been described include atherosclerosis, myocardial infarction, and cardiac arrhythmias (such as atrial fibrillation or atrioventricular block), whereas a higher prevalence of celiac disease has been described in patients with dilated cardiomyopathy, myocarditis, and pericardial effusion [7]. However, the cardiovascular risk in adult celiac disease patients remains controversial [8]. On the other hand, recent studies based on advanced echocardiography demonstrate the subclinical ventricular dysfunction of both the left and right ventricle. Considering the limited data on pediatric patients, the contradictory scientific evidence on adults and the low anticipated prevalence of cardiovascular events at a young age, we conducted a systematic review and meta-analysis in order to investigate the subclinical effect of celiac disease on myocardial function. Finally, as it remains unclear whether adherence to a gluten-free diet (GFD) can reduce the risk of myocardial dysfunction among pediatric patients, we also assessed whether GFD is associated with cardiac function improvement.

## 2. Methods

### 2.1. Search Methodology

This systematic review and meta-analysis was carried out following the Preferred Reporting Items for Systematic Reviews and Meta-Analyses (PRISMA) guidelines. A prespecified protocol has been registered in PROSPERO (ID: CRD42024512242). The search strategy was based on original articles in the main medical databases (PubMed/MEDLINE, Scopus) reporting the cardiac assessment of pediatric and adolescent celiac disease patients, including the relevant terms for celiac disease, cardiac dysfunction, and children. To this end, the following search terms were used for PubMed/MEDLINE and Scopus:

((celiac disease) OR (coeliac disease) OR (gluten)) AND ((myocardi*) OR (cardi*) OR (heart) OR (ventric*) OR (echo*) OR (doppler)) AND ((child*) OR (pedi*) OR (adolesc*))
  (ALL (“celiac disease”) AND ALL (myocardi* OR card* OR heart OR ventric*) AND ALL (child* OR pediatric* OR adolescen*) AND ALL (echo* OR doppler)) AND (LIMIT-TO (SUBJAREA, “MEDI”))

The last search was conducted on 3 March 2024; thus, studies published on or before 3 March 2024 were included in the meta-analysis. Although a systematic literature search was conducted through the major electronic databases up to 3 March 2024, an extended search for articles published in the last year, using the same search terms as in the initial literature review, did not identify additional studies that met our strict inclusion criteria. Consequently, it appears that the data included in the current review provide a robust and comprehensive representation of the available evidence up to February 2025. There was no limitation for publication year. The references of the selected articles were further screened to identify additional articles. PROSPERO, Clinicaltrials.gov, OSF, Cochrane Database of Systematic Reviews, ResearchGate’s search engine, Google Scholar, conference papers, and gray literature were searched for duplicates or potentially relevant studies, abstracts, or trials (published or not). The literature review was independently performed by two reviewers, and any disagreement regarding study inclusion was resolved by consensus. This study included only articles published in the English language.

### 2.2. Eligibility Criteria

We included observational studies (case-control or cohort), reporting echocardiographic parameters of myocardial function in children and adolescent patients with celiac disease. We included case–control studies investigating celiac disease patients compared to matched controls or cohort studies with groups of those exposed and unexposed to celiac disease. The following criteria were used for study eligibility: (1) Studies with participants’ age under 18 years; (2) Biopsy-proven diagnosis of celiac disease or based on ESPGHAN criteria [9]; (3) Studies reporting parameters based on conventional and advanced echocardiography. The exclusion criteria were the following: (1) Studies without a control arm; (2) Studies reporting undiagnosed celiac disease; (3) Studies not reporting echocardiographic parameters; (4) Studies in adults (over 18 years of age) or unclear participants’ age; (5) Not extractable data.

### 2.3. Study Procedure and Data Extraction

The literature search was conducted independently by two authors, and the records were imported using a reference management tool (www.rayyan.ai) accessed on 3 March 2024. Duplicates were removed, and two authors independently screened the titles and abstracts of all the retrieved articles. Studies deemed potentially eligible were then retrieved in full, and they were independently assessed through full-text reading. Any disagreement was resolved with a third reviewer who made the final decision. The following data were independently extracted from the included studies into a prespecified form: (1) publication year; (2) study design and location; (3) study population and number of patients included in each arm; (4) duration of follow-up; (5) rates of dietary adherence to GFD in celiac disease patients, if feasible; and (6) echocardiographic parameters.

### 2.4. Quality Assessment

Assessment of the risk of bias (RoB) in the included studies was conducted by two reviewers independently, using the Quality in Prognosis Studies (QUIPS) tool [10]. This tool was developed to assess the risk of bias in the results of non-randomized studies, recommended by the Cochrane Prognosis Methods Group. QUIPS consists of six important domains: (1) The risk of selection bias; (2) The risk of attrition bias; (3) The risk of measurement bias related to how the prognostic factor is measured; (4) The risk of bias related to the measurement of the outcome; (5) Bias due to confounding; (6) Bias related to statistical analysis and presentation of the results. Studies were classified as low risk, if only up to one domain was considered moderate and as high risk if at least one domain was considered high or three or more domains were considered moderate [11]. The visualization of the assessment was performed using the robvis tool [12]. Any disagreements were resolved with the final decision of a third senior reviewer.

### 2.5. Echocardiographic Parameters

Echocardiographic parameters, both conventional and advanced, were used to assess and define subclinical myocardial function. Ejection fraction (EF) and fractional shortening (FS), reflecting left ventricular systolic function, were estimated by conventional echocardiography. Moreover, the right and left ventricular myocardial performance index (RV and LV MPI), also known as the TEI index, which reflects global ventricular function, was assessed by advanced echocardiography. Finally, mitral and tricuspid valve E/E’ ratios were measured for the assessment of diastolic function. For each outcome, the mean value and standard deviation (SD) were extracted for each arm. In the case of myocardial dysfunction, the expected echocardiographic changes included reduced EF and FS (indicating reduced systolic function), increased RV and LV MPI (indicating impaired myocardial function), and higher mitral and tricuspid valve E/E’ ratios (indicating diastolic dysfunction) [13]. A secondary outcome was the potential effectiveness of GFD in improving myocardial function, assessed by the echocardiographic parameters mentioned above.

### 2.6. Statistical Analysis

R version 4.2.3 (2023) for Windows (R Core Team, Vienna, Austria) was used for the statistical analysis of the meta-analysis. At least three studies were required to combine each of the above-mentioned echocardiographic parameters on pooled outcomes. The descriptive measures of continuous data were the mean value and standard deviation (SD) or the median and interquartile range (IQR), based on the distribution of each parameter. Summary estimates were calculated, using the mean difference (MD) with SD and a 95% confidence interval (CI). Heterogeneity between studies was assessed using both the χ^2^ test and the I^2^ statistic. A high anticipated heterogeneity between studies arose from differences in the centers and populations, study designs, mean follow-up time of patients, and variable adherence to gluten-free diet rates among studies. While an I^2^ value greater than 50% was indicative of substantial heterogeneity, it was the inherent differences between the studies that led us to consider more appropriate random effect models for the analysis, using the inverse variance method. A leave-one-out analysis (omitting each study consecutively to investigate its effect on the overall outcome) and Egger’s test were conducted, in order to assess evidence of publication bias. A sensitivity analysis was conducted in order to compare the echocardiographic parameters of children and adolescents with celiac disease, classified into two groups: those with a strict adherence to GFD (negative serum antibodies) and those with positive serologic markers, indicative of a poor adherence to GFD. When necessary, data from multiple subgroups (i.e., treated and untreated celiac patients) were combined into a single group (i.e., celiac disease patients) using the *n*, mean, and SD based on Cochrane’s formula [14] (i.e., treated and untreated celiac patients combined into a single group in order to compare their echocardiographic parameters to those of healthy children). A two-sided *p*-value threshold of 0.05 determined statistical significance for all analyses.

## 3. Results

### 3.1. Search Results

**Figure 1:**
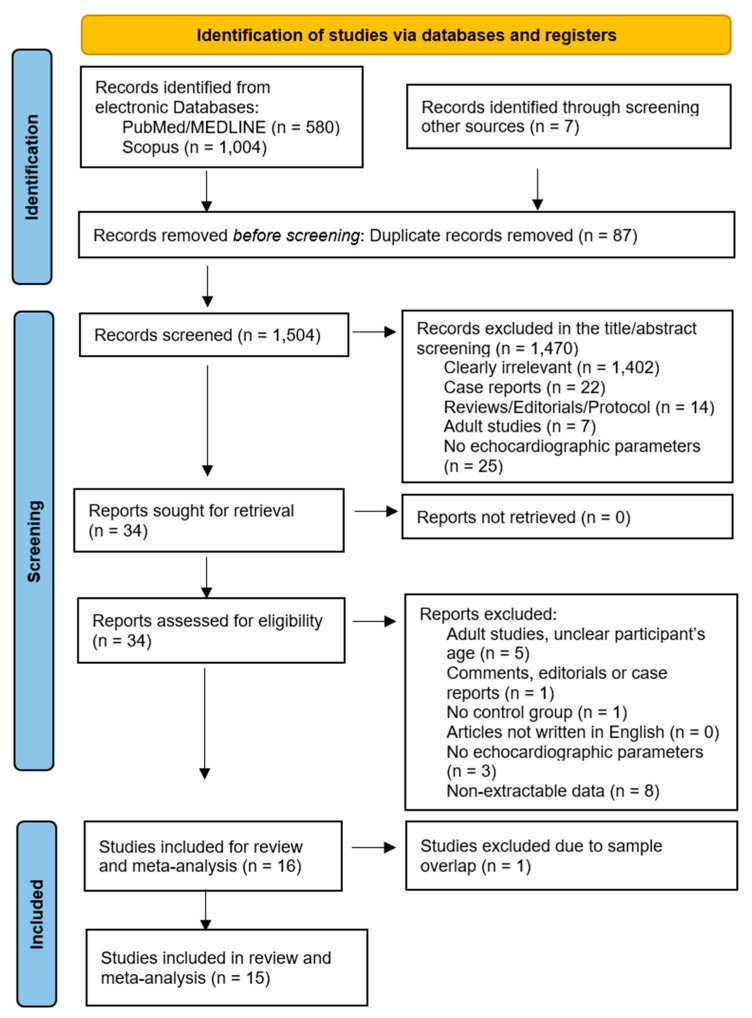
PRISMA 2020 flow chart of the included studies.

The study selection flowchart is presented in Figure 1. Initially, 1591 records were identified in total, and after duplicate removal, 1504 studies remained. Of these, 1470 were excluded as irrelevant, based on abstract or title screening. Full-text access was available for all remaining 34 studies, and another 18 out of the 34 studies were further removed following the exclusion criteria. Therefore, 16 studies were eligible for the meta-analysis, and after the exclusion of one study [15] due to sample overlap, 15 studies were included. The echocardiographic parameters measured from each study (mean value and standard deviations) are provided separately in Supplementary Table S1.

### 3.2. Characteristics of the Included Studies

The characteristics of the 15 selected studies are provided in Table 1. A total of 916 children and adolescents with celiac disease were included in the study. Eight studies incorporated two separate groups of celiac disease patients based on adherence to GFD. The dietary compliance evaluation was based on serum IgA-tissue transglutaminase (IgA-tTG) or anti-endomysial (EMA) antibodies, as indicated in Table 1. In 13 out of the 15 studies, tissue Doppler echocardiography was used to assess myocardial function. The echocardiographic parameters measured from each study (mean value and standard deviations) are provided in Supplementary Table S1.

### 3.3. Risk of Bias in the Included Studies

**Figure 2:**
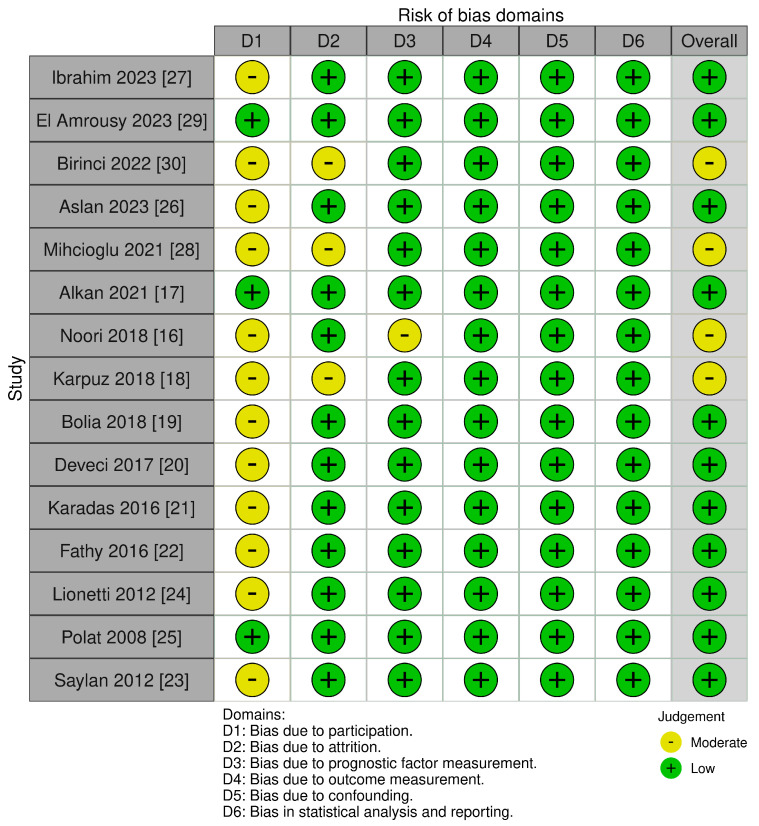
Risk of bias plot.

A risk of bias summary assessment is described in Figure 2. The majority of studies were considered as “low” risk, whereas only four studies were graded as “moderate”, based on the Quality in Prognostic Studies (QUIPS) tool [10].

### 3.4. Conventional and Tissue Doppler Echocardiographic Parameters in Children and Adolescents with Celiac Disease

**Figure 3:**
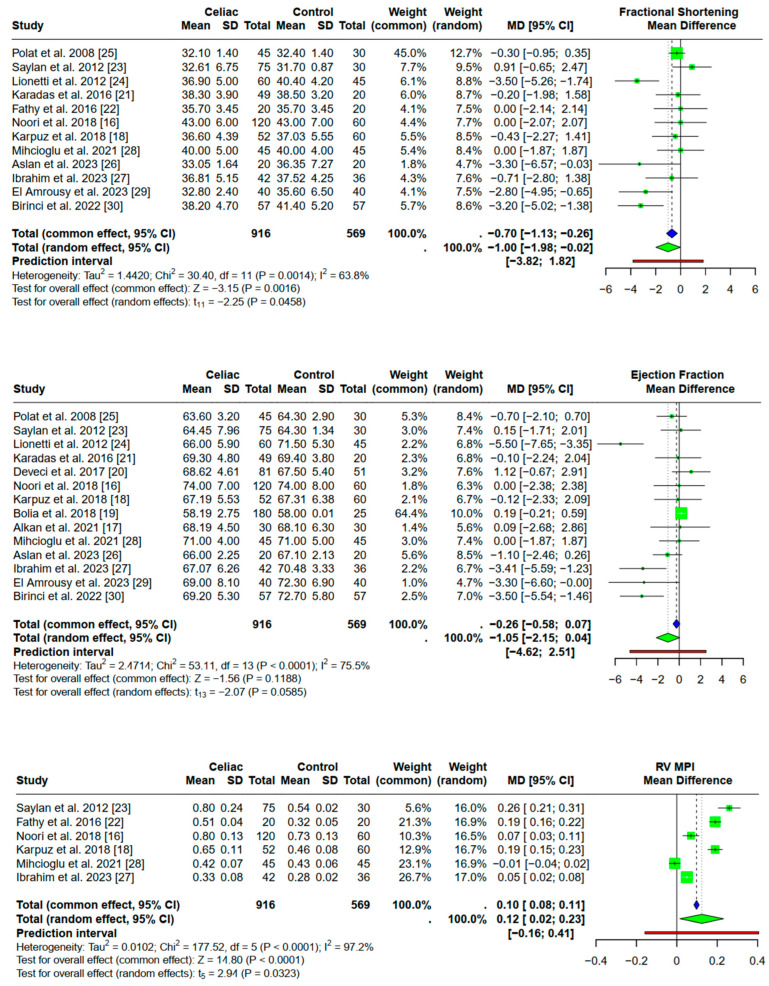

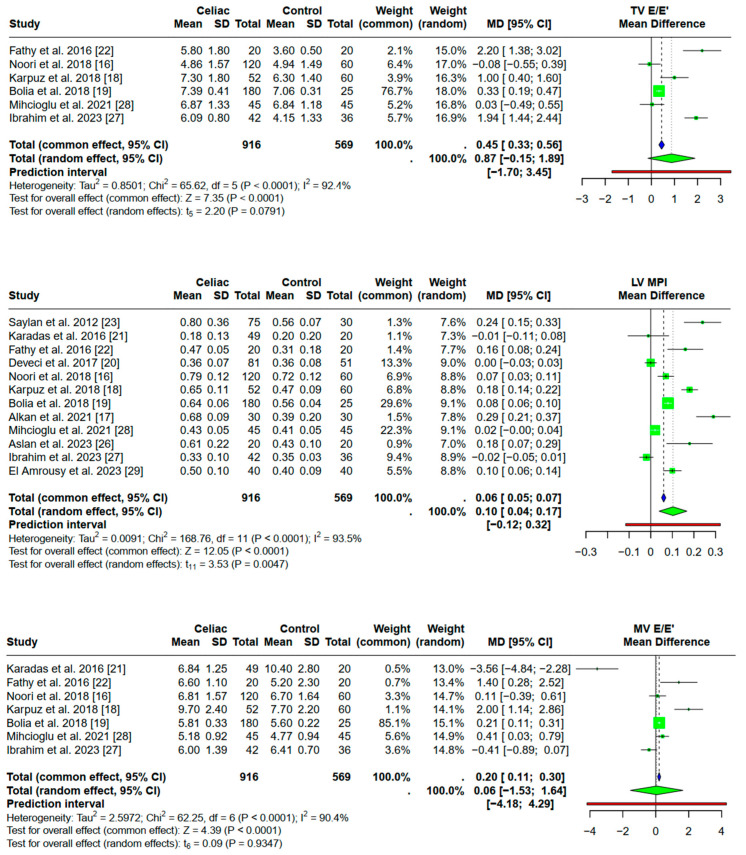
Forest plots of conventional and tissue Doppler echocardiographic parameters. This dot represents NA (not applicable).

The forest plots in Figure 3 illustrate the pooled effect sizes for all the included studies, showing the degree of heterogeneity and the effect of celiac disease on each echocardiographic parameter. The size of the squares indicates the weight of each study, and the horizontal lines represent the 95% confidence intervals. In the majority of studies, there is a positive association between celiac disease and myocardial dysfunction (as determined by the mean difference of each echocardiographic parameter between celiac patients and controls).

More specifically, a collection of data from 12 studies revealed that celiac disease was associated with a mean decrease in fractional shortening of 1% (95% CI [−1.98; −0.02]) (*p* = 0.05). Regarding the EF, we collected data from 14 studies. Although EF was lower in patients compared to controls, the pooled effect was marginally not statistically significant (*p* = 0.06). The mean difference was −1.05% (95% CI −2.15; 0.04). Based on the pooled effect of six available studies, celiac disease patients had a statistically significant higher RV MPI index compared to controls (*p* = 0.03). The mean difference was 0.12 with a 95% CI of [0.02; 0.23]. According to the random effect models, TV E/E’ was not statistically increased in patients with celiac disease (*p* = 0.08). A collection of data from 12 studies showed a higher LV MPI index in patients with celiac disease compared to controls (*p* < 0.01). LV MPI was found to be 0.10 higher in patients, 95% CI [0.04; 0.17]. Finally, MV E/E’ did not differ between the two groups (*p* = 0.93).

### 3.5. Effect of GFD Adherence on Echocardiographic Parameters

**Figure 4:**
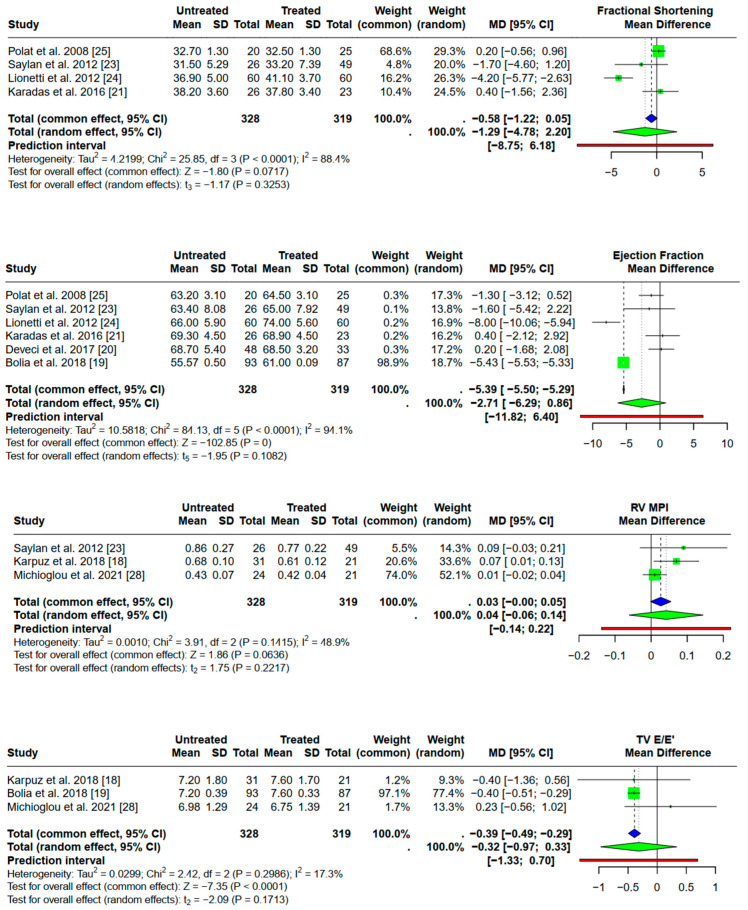

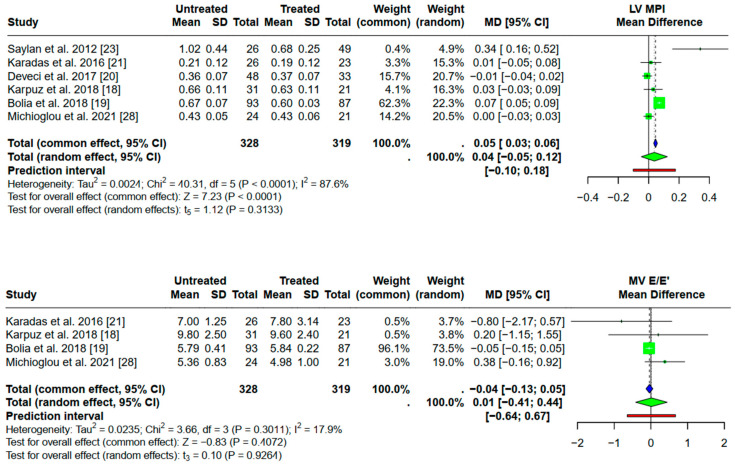
Forest plots of echocardiographic parameters after adherence to GFD. This dot represents NA (not applicable).

Figure 4 presents the forest plots comparing subgroup analyses, highlighting differences in effect sizes between compliant and non-compliant GFD patients. After adherence to a gluten-free diet, there was no significant increase either in FS (data from four collected studies) or in EF (data from six collected studies) (*p* = 0.33 and *p* = 0.11, respectively), although the pooled FS effect was 1.29 (95% CI [−4.78; 2.20] and EF 2.71 (95% CI [−6.29; 0.86] lower in patients who never received GFD.

In addition, adherence to a gluten-free diet did not have any impact on the RV MPI index (*p* = 0.22, pooled effect of three available studies) or the LV MPI index (*p* = 0.31, data collection of six studies). Finally, according to the random effect models, TV E/E’ and MV E/E’ did not differ statistically between celiac disease patients who were on GFD (*p* = 0.17 and *p* = 0.93, respectively).

### 3.6. Leave-One-Out Analysis

A leave-one-out sensitivity analysis was performed for each echocardiographic parameter, by sequentially removing each study at a time from the overall analysis, in order to investigate the existence of any significant effect on the pooled estimate.

### 3.7. Publication Bias Assessment

An asymmetrical funnel plot and Egger’s test were used to assess publication bias [31], although the statistical power of these tests may not be high enough to detect real asymmetry in small samples. Based on the visual interpretation of the funnel plots and the results from the Egger’s regression test, we determined that there was no significant indication of publication bias among the studies included in this meta-analysis.

## 4. Discussion

The results of our systematic review reveal a growing body of evidence of the effects on myocardial function in children and adolescents with celiac disease. More specifically, our results indicate the presence of potential subclinical myocardial dysfunction in pediatric and adolescent celiac disease patients; using advanced echocardiographic techniques, we showed that the pooled RV and LV MPIs were significantly higher in pediatric patients compared to controls.

The existing literature related to myocardial function in children and adolescents with celiac disease is mainly based on conventional echocardiographic parameters. However, the use of advanced echocardiographic techniques, such as the myocardial performance index (MPI) and, less often, global longitudinal strain (GLS), was also assessed in the studies included in this review.

In our study, FS was lower in pediatric celiac disease patients compared to controls. The other parameters studied did not differ significantly. Although EF tended to be higher and tricuspid valve E/E’ lower in healthy individuals, the pooled effects were marginally not statistically significant. Furthermore, there was no statistical difference in the mitral valve E/E’ ratio between patients and controls. The discrepancies between echocardiographic parameters indicate that MPI, measured by advanced echocardiography, is an earlier marker of myocardial dysfunction than EF and E/E’. However, conventional echocardiography should not be underestimated because it is a useful non-invasive tool for assessing myocardial function. Even if differences between diastolic dysfunction markers were not established, there was a trend toward reduced myocardial function in all echocardiographic parameters.

The myocardial performance index, also known as the Tei index, is considered a non-invasive parameter that may offer valuable insights into myocardial function in adults. It provides useful information about the overall function of the myocardium that can reveal myocardial abnormalities before they are detected by conventional echocardiography (EF or FS) [13]. In adults, a higher MPI value indicates poorer cardiac function in various heart diseases, such as heart failure and cardiomyopathy. The use of MPI in children and adolescents is still under investigation, and no established normal values for this group exist, limiting the wide use of MPI in daily clinical practice. Although the relationship between MPI and clinical outcomes in this population requires further validation, higher MPI values, even in children, are often associated with impaired myocardial function [13]. Finally, in our meta-analysis, although MPI does not seem to be a clinically attractive parameter, the comparison between patients and controls is valuable, incontrovertible, and enhances interpretability, especially in cases where a non-invasive assessment is required.

In recent years, global longitudinal strain (GLS) has become an emerging method, which has been considered in adults as an early marker of myocardial dysfunction. Clinical utility in children is limited, and regarding celiac disease, only two studies have been published so far [20,29]. However, both studies report an impaired GLS, indicating early myocardial changes and subclinical left ventricular dysfunction. Although the results of only two studies could not be combined into pooled effects, an impaired GLS reflects an early impairment of myocardial function. We anticipate that future studies that include advanced imaging techniques, such as GLS, will establish more robust evidence for the clinical utility on celiac disease pediatric patients.

Our results also indicate the presence of impaired ventricular function in children and adolescents with untreated celiac disease. A possible negative effect of celiac disease on cardiovascular function has been documented so far in the literature, with studies correlating celiac disease with dilated cardiomyopathy in adults [7,8]. Although the pathogenic mechanism needs further investigation, a reasonable thought is that as celiac disease is an auto-immune disease, auto-immunity is responsible for early myocardial injury.

Finally, our results showed that there was a lack of significant improvement in echocardiographic parameters after adherence to GFD. A possible explanation could be that the myocardium is exposed to auto-antibodies for a long period of time and needs more time to reverse damage. Nevertheless, the timing of the echocardiographic examination in the included studies is an important consideration that may influence the observed findings in both adherent and non-adherent patients. It is reasonable to assume that the effects on the cardiovascular system may differ depending on the stage of the disease, including factors such as recent diagnosis, long-term adherence to a gluten-free diet, recent normalization of antibodies, and non-adherence to a gluten-free diet over an extended period. Therefore, prospective studies with a long-term follow-up are needed in order to compare possible myocardial improvement with adherence to GFD. On the other hand, myocardial injury could be related to other malnutrition abnormalities, such as anemia, low albumin, lipid abnormalities, etc., that play a vital role as independent cardiometabolic risk factors. So, the normalization of any nutritional deficiency or lipid abnormality should be considered before assessing the efficacy of GFD.

In summary, this meta-analysis indicates that celiac disease has systemic implications beyond the gastrointestinal system, which involve the cardiovascular system, that can even start in childhood. In addition, our study suggests that the cardiovascular system should not be overlooked and underestimated in pediatric celiac disease patients and underscores the importance of monitoring cardiac function. Though not clinically evident, our meta-analysis describes an early myocardial abnormality as reflected by higher MPI values. However, further research is needed, as studies in adults have shown the association of celiac disease not only with cardiovascular diseases (myocardial infraction, atrial fibrillation, myocarditis, cardiomyopathy, and stroke) [32,33,34] but also with cardiovascular risk factors [8,35]. Nutritional deficiencies, chronic inflammation leading to atherosclerosis, and autoimmunity (such as an increased risk of type 1 diabetes) [19,36] may result in an increased risk of cardiovascular diseases, although individuals with celiac disease have lower rates of conventional risk factors (such as smoking or elevated BMI) [35,37]. Thus, in order to prevent the long-term consequences, children and adolescents with celiac disease should be regularly followed up for the early detection of subclinical cardiac abnormalities.

The main advantage of this meta-analysis is the large number of included studies and the variety of echocardiographic parameters. Covering a total sample of 916 celiac disease patients and 569 healthy controls, this meta-analysis provides high statistical power and greater precision in the estimation of the actual effect size. Furthermore, by incorporating diverse populations into random effect models, the meta-analysis provides generalizable results. Finally, the prespecified protocol registered in PROSPERO ensures a systematic and transparent methodology enabling more reliable conclusions.

Our systematic review and meta-analysis also has some limitations that must be acknowledged. To begin with, the method of adherence to GFD was not consistent. In addition, the rate of dietary adherence and the mean time since diagnosis were not described in all the included studies. To this end, random effect models were primarily taken into consideration to estimate the pooled effect and ensure the validity of the conclusions drawn from the meta-analysis. Moreover, not all studies assessed the same echocardiographic parameters, narrowing the total sample available for each estimate. However, more than three studies were available in order to combine the data into pooled effects. Another limitation of this meta-analysis is that it does not include the pooled effects of global longitudinal strain (GLS) as a parameter of early myocardial dysfunction. Although GLS was not excluded from our broad search strategy, we found limited studies specifically investigating GLS in the context of myocardial function in children and adolescents with celiac disease [20,29]. The results of only two studies were available, and these could not be combined into pooled effects. However, this remains an interesting goal for future research as the evolving role of GLS in clinical practice highlights the need for more multicenter studies to establish normal reference values for GLS in childhood in order to be incorporated in clinical practice as a useful noninvasive tool. As GLS data become more widely available in future studies, robust and reliable pooled effects will be provided. MPI was used in this meta-analysis based on previous studies with sufficient for statistics data. However, both parameters should be used to assess early myocardial function in children. We would also like to address the lack of the z-scores of the echocardiographic parameters in the original studies. Since z-scores account for age- or size-related variations in cardiac parameters, they provide a more accurate standardization for interpretation across different age groups. To address this issue, most studies incorporated these variations into their study design by matching the control group for parameters such as age, sex, body surface area, or BMI (Table 1). Therefore, we believe that the lack of z-scores does not reduce the quality or precision of our results. Furthermore, in order to address the sample overlap between two studies from the same center [15,16] without inflating the precision of estimates, we excluded upon consensus the one with the smallest number of participants, as currently there is no standard methodological approach to deal with the overlap of primary studies in reviews and meta-analyses. It is noteworthy that there is a profound representation of Turkey among the included studies. Comparable to global levels cited as around 1% [3], Turkey does not exhibit an unusually high prevalence of celiac disease compared to other Eastern or Mediterranean regions [38]. Thus, based on the regional variation of celiac disease prevalence [3], more studies from European countries would be anticipated. Moreover, the observed differences are small and may be considered clinically inconclusive and not useful in clinical practice, highlighting the need for further investigation before drawing definitive clinical conclusions. In addition, each echocardiographic parameter should be considered and utilized on a case by case basis in clinical practice.

## 5. Conclusions

Our findings underscore the vital importance of the early myocardial assessment of children and adolescents with celiac disease, even in the absence of clinical indications. The early recognition of possible myocardial dysfunction, as well as the identification of other risk factors, combined with a strict adherence to GFD can mitigate long-term complications and improve health outcomes in pediatric celiac disease patients.

Therefore, an examination of the cardiovascular system should be incorporated into the regular follow-up of children and adolescents with celiac disease in order to detect early subclinical myocardial dysfunction. MPI is suggested as a reliable, non-invasive marker for assessing myocardial function in pediatric celiac disease patients.

## Figures and Tables

**Table 1:** Baseline characteristics of all the included studies.

Study ID	Study Duration	Patients	Mean Follow-Up/Compliance Rate	Controls	Exclusion Criteria
Noori [16] 2018 Iran	August 2015 to July 2016	120 children with biopsy-proven celiac disease		60 healthy children (1–18 years old) in a single center, matched for sex and age	Patients with valvular disease, rhythm abnormality, CHD, malignancy, systemic inflammatory diseases, diabetes mellitus, renal insufficiency, chronic obstructive pulmonary disease, and hypertension
Alkan [17] 2021 Turkey	25 July 2016 to 25 July 2017	30 children (4–18 years old)	80%	30 healthy children matched for age and gender, admitted to the pediatric cardiology outpatient clinic due to other reasons	Patients with systemic hypertension, diabetes mellitus, presence of other systemic diseases, primary valve insufficiency, genetic cardiomyopathy, peripartum cardiomyopathy, chronic infection or known toxin-related cardiomyopathy, structural heart disease, coronary artery disease, collagen tissue disease, hematological disease, renal/hepatic failure, malignancy, and patients who did not give their consent
Karpuz [18] 2018 Turkey		Children (under 18 years old) with biopsy-proven celiac disease: Group 1: IgA-tTG positive (*n* = 31) Group 2: IgA-tTG negative (*n* = 21)	Mean follow-up time 4.20 ± 2.12 years	60 healthy, age-, gender-, and body-surface- area-matched children with normal findings on clinical examination	Not mentioned
Bolia [19] 2018 India	January 2013 to January 2015	**Group 1:** 40 newly diagnosed children (age 4.2 ± 1.1 years) that were compliant to GFD (based on tTG) at the end of the follow-up period **Group 2**: 100 children on GFD (age 8.6 ± 3.4 years); 47 compliant (mean age 7.9 ± 2.7 years) vs. 53 non-compliant based on tTG (mean age 10.8 ± 1.9)	**Group 1:** 16 (12–21) months **Group 2:** 3.2 ± 2.1 years after starting GFD	25 age- and sex-matched healthy controls	Children with underlying cardiovascular illness, pulmonary disease, or cardiac malformations
Deveci [20] 2017 Turkey	Not mentioned	81 children **divided into two groups:** Group 1: IgA-tTG positive (*n* = 48) Group 2: IgA-tTG negative (*n* = 33)	More than 6 months on GFD	51 healthy children matched for age, sex, and body mass index	Patients with known active inflammation, structural/valvular/congenital heart disease, hypertension, malignancy, anemia, thyroid dysfunction, or any other chronic diseases
Karadas [21] 2016 Turkey	July 2012 to January 2015	49 patients Group 1: 26 (53%) patients not on GFD Group 2: 23 (47%) patients on GFD for at least 10 months	Average follow-up period for Group 2: 36.3 ± 25.2 months (range: 10–126)	20 healthy children	Not mentioned
Fathy [22] 2016 Saudi Arabia	February 2015 and August 2015	20 patients with biopsy-proven celiac disease: 13 patients not on GFD and 7 non-compliant and still symptomatic patients	Mean follow-up of 7 non-compliant patients: 32.2 ± 17.1 months	20 age- and sex-matched healthy children	Pre-existing cardiac disorders or medication affecting the cardiac function. Additionally, any gastrointestinal problems for healthy children
Saylan [23] 2012 Turkey	May 2009 to June 2010	75 children (mean age 9.3 ± 4.6 years; range 5 months–19 years) with biopsy-proven celiac disease Group 1: EmA positive (*n* = 26) Group 2: EmA negative (*n* = 49)	Mean follow-up time 34 ± 15.1 months Group 1: 34.2 ± 28.1 months Group 2: 20.6 ± 18.8 months	30 age-matched healthy children	Not mentioned
Lionetti [24] 2012 Italy	November 2008 to June 2009	60 children with biopsy-proven celiac disease (mean age 5.5 years; range 4.6, 6.4) re-evaluated after 1-year GFD	At diagnosis and after 12 months of GFD	45 healthy children Age/gender/BMI matched, living in the same geographic area, mean age 6.9; range 5.5–8.3	Any congenital or acquired heart disease, other known diseases, and the use of medication that can modify cardiac function Controls: no disease or previous positive medical history, or family history of celiac/heart disease
Polat [25] 2008 Turkey	Not mentioned	45 patients (mean age 10.8 ± 4.5 years; range 2–19 years) **Group 1:** IgA EMA (+) (*n* = 25) **Group 2**: IgA EMA (−) (*n* = 20)	Mean follow-up time 32.2 ± 17.1 months (6–60 months)	30 healthy, matched for age children, tested negative for serum EMA	
Aslan [26] 2023 Egypt	From December 2021 to December 2022	20 patients (mean age 8.65 ± 3.65 years); 57.5% newly diagnosed, 31.4% on non-strict GFD and 11.1% on strict GFD	Mean duration of celiac disease: 14.4 ± 10.73 months	20 healthy, age- and sex-matched children (mean age 9.24 ± 2.39 years)	Pre-existing cardiac conditions, taking any substances that might impact heart function, long-term illnesses that affect heart function (systemic lupus erythematosus, chronic renal disease, or diabetes mellitus)
Ibrahim [27] 2023 Egypt	From December 2021 to May 2022	42 patients with biopsy-proven celiac disease (mean age 8.8 ± 3.8 years)	6 months after strict GFD; 2 (4.8%) non-compliant	36 healthy, age- and sex-matched children	Celiac disease: other gastrointestinal illnesses, congenital or acquired heart diseases, and additional systemic illnesses, e.g., diabetes mellitus and autoimmune thyroiditis Controls: gastrointestinal or cardiac problems
Mihcioglou [28] 2021 Turkey	Not mentioned	45 biopsy-proven patients (mean age 10.70 ± 4.40 years) **Group 1:** IgA tTG and EMA (+) (*n* = 24) **Group 2**: IgA tTG and EMA (−) (*n* = 21)	Mean duration of celiac disease: 2.47 years (0.25–8 years)	45 patients; mean age 10.2 ± 2.6 years	Controls: murmur without any cardiac disorder
El Amrousy [29] 2023 Egypt	December 2021 to December 2022	40 children; mean age 8.5 ± 2.4 years	All patients were on GFD, with mean duration of the disease 16.2 ± 7.5 months; **Dietary compliance rate of patients 80%**	40 healthy, sex- and age- matched children	Children with pre-existing cardiac conditions, systemic disorders (such as hypertension, diabetes mellitus, or thyroid dysfunction), or taking any medication that may affect cardiac function
Biricini [30] 2022 Turkey	Not mentioned	57 celiac patients (mean age 10.5 ± 4.2 years) tTG-IgA positive 64.9% (*n* = 37) tTG-IgA negative 35.1% (*n* = 20) EMA-IgA positive 57.9% (*n* = 33) EMA-IgA negative 42.1% (*n* = 24)		Healthy children of similar age and gender to the patient group, who performed a pre-participation physical examination prior to participation in sports; mean age 10.4 ± 3.9 years.	Celiac disease: additional chronic diseases Controls: celiac disease, or additional chronic diseases

## Data Availability

The data used in this meta-analysis were extracted from publicly available studies, which are cited in the manuscript. All data sources, including the study identifiers, are listed in the references. Furthermore, the data supporting the findings of this meta-analysis are available from the corresponding author upon reasonable request.

## Supplementary Materials

The following supporting information can be downloaded at: https://www.mdpi.com/article/10.3390/children12040441/s1, Table S1: Echocardiographic parameters of the included studies.

## Abbreviations

The following abbreviations are used in this manuscript:

GFD	Gluten-free diet
FS	Fractional shortening
EF	Ejection fraction
LV MPI	Left ventricle myocardial performance index
RV MPI	Right ventricle myocardial performance index
TV	Tricuspid valve
MV	Mitral valve
