# Supplementary material for: Subclinical Myocardial Dysfunction in Pediatric and Adolescent Celiac Disease Patients: A Systematic Review and Meta-Analysis
Source: Children (Basel). 2025 Mar 30;12(4):441. doi: 10.3390/children12040441 (PMC12025519; doi:10.3390/children12040441)
Supplement: Supplementary file 1 [file children-12-00441-s001.zip › children-3445453-supplementary.pdf]

Supplementary Table S1. Echocardiographic parameters of the included studies.

Study ID	Cardiovascular evaluation	Echocardiographic Parameter	Celiac Mean (SD)	Control Mean (SD)	Treated CD Mean (SD)	Untreated CD Mean (SD)
Noori **[16]** 2018 Iran	ECG, conventional transthoracic echocardiography, and tissue Doppler echocardiography	FS	43 (6)	43 (7)	-	-
		EF	74 (7)	74 (8)	-	-
		RV MPI	0.8 (0.13)	0.73 (0.13)	-	-
		TV E/E′	4.86 (1.57)	4.94 (1.49)	-	-
		LV MPI	0.79 (0.12)	0.72 (0.12)	-	-
		MV E/E′	6.81 (1.57)	6.7 (1.64)	-	-
Alkan **[17]** 2020 Turkey	Conventional transthoracic echocardiography, tissue Doppler imaging, and aortic elasticity	FS	-	-	-	-
		EF	68.19 (4.5)	68.1 (6.3)	-	-
		RV MPI	-	-	-	-
		TV E/E′	-	-	-	-
		LV MPI	0.68 (0.09)	0.39 (0.2)	-	-
		MV E/E′	-	-		
Karpuz **[18]** 2018 Turkey	Conventional transthoracic echocardiography, tissue Doppler imaging, and aortic elasticity	FS	36.60 (4.39)	37.03 (5.55)	-	-
		EF	67.19 (5.53)	67.31 (6.38)	-	-
		RV MPI	-	0.46 (0.08)	0.61 (0.12)	0.68 (0.1)
		TV E/E′	-	6.3 (1.4)	7.6 (1.7)	7.2 (1.8)
		LV MPI	-	0.47 (0.09)	0.63 (0.11)	0.66 (0.11)
		MV E/E′	-	7.7 (2.2)	9.6 (2.4)	9.8 (2.5)
Bolia **[19]** 2018 India	Conventional transthoracic echocardiography and tissue Doppler imaging	FS	-	-	-	-
		EF	-	58 (0.01)	57 (0.04)	55 (0.07)
		RV MPI	-	-	-	-
		TV E/E′	-	7.06 (0.31)	6.96 (0.11)	7.43 (0.37)
		LV MPI	-	0.56 (0.04)	0.57 (0.11)	0.69 (0.04)
		MV E/E′	-	5.6 (0.22)	5.5 (0.22)	5.9 (0.4)
Deveci **[20]** 2017 Turkey	Conventional transthoracic echocardiography, tissue Doppler imaging, and two-dimensional speckle-tracking echocardiography	FS	-	-	-	-
		EF	-	67. (5.4)	68.5 (3.2)	68.7 (5.4)
		RV MPI	-	-	-	-
		TV E/E′	-	-	-	-
		LV MPI	-	0.36 (0.08)	0.37 (0.07)	0.36 (0.07)
		MV E/E′	-	-	-	-
Karadas **[21]** 2016 Turkey	Conventional transthoracic echocardiography and tissue Doppler imaging	FS	38.3 (3.9)	38.5 (3.2)	37.8 (3.4)	38.2 (3.6)
		EF	69.3 (4.8)	69.4 (3.8)	68.9 (4.5)	69.3 (4.5)
		RV MPI	-	-	-	-
		TV E/E′	-	-	-	-
		LV MPI	0.18 (0.13)	0.12 (0.2)	0.19 (0.12)	0.21 (0.12)
		MV E/E′	6.84 (1.25)	10.4 (2.8)	7.8 (3.14)	7 (1.25)
Fathy **[22]** 2016 Saudi Arabia	Conventional transthoracic echocardiography and tissue Doppler imaging	FS	35.7±3.45	35.7±3.45	-	-
		EF	-	-	-	-
		RV MPI	0.51 (0.04)	0.32 (0.05)	-	-

		TV E/E′	5.8 (1.8)	3.6 (0.5)	-	-
		LV MPI	0.47 (0.05)	0.31 (0.18)	-	-
		MV E/E′	6.6 (1.1)	5.2 (2.3)	-	-
Saylan **[23]** 2012 Turkey	Conventional transthoracic echocardiography and tissue Doppler imaging	FS	-	31.7 (0.87)	33.2 (7.39)	31.5 (5.29)
		EF	-	64.3 (1.34)	65.0 (7.92)	63.4 (8.08)
		RV MPI	-	0.54 (0.02)	0.77 (0.22)	0.86 (0.27)
		TV E/E′	-	-	-	-
		LV MPI	-	0.56 (0.07)	0.68 (0.25)	1.02 (0.44)
		MV E/E′	-	-	-	-
Lionetti **[24]** 2012 Italy	Conventional transthoracic echocardiography	FS	36.9 (5)	40.4 (4.2)	41.1 (3.7)	36.9 (5)
		EF	66 (5.9)	74 (5.3)	74 (5.6)	66 (5.9)
		RV MPI	-	-	-	-
		TV E/E′	-	-	-	-
		LV MPI	-	-	-	-
		MV E/E′	-	-	-	-
Polat **[25]** 2007 Turkey	Conventional transthoracic echocardiography and tissue Doppler imaging	FS	32.1 (1.4)	32.4 (1.4)	32.5 (1.3)	32.7 (1.3)
		EF	63.6 (3.2)	64.3 (2.9)	64.5 (3.1)	63.2 (3.1)
		RV MPI	-	-	-	-
		TV E/E′	-	-	-	-
		LV MPI	-	-	-	-
		MV E/E′	-	-	-	-
Aslan **[26]** 2023 Egypt	Conventional transthoracic echocardiography and tissue Doppler imaging	FS	33.05 (1.64)	36.35 (7.27)	-	-
		EF	66 (2.25)	67.1 (2.13)	-	-
		RV MPI	-	-	-	-
		TV E/E′	-	-	-	-
		LV MPI	0.61 (0.22)	0.43 (0.1)	-	-
		MV E/E′	-	-	-	-
Ibrahim **[27]** 2023 Egypt	Conventional transthoracic echocardiography and tissue Doppler imaging	FS	36.81 (5.15)	37.52 (4.25)	-	-
		EF	67.07 (6.26)	70.48 (3.33)	-	-
		RV MPI	0.33 (0.08)	0.28 (0.02)	-	-
		TV E/E′	6.09 (0.8)	4.15 (1.33)	-	-
		LV MPI	0.33 (0.10)	0.35 (0.03)	-	-
		MV E/E′	6 (1.39)	6.41 (0.7)	-	-
Mihcioglou **[28]** 2021 Turkey	Conventional transthoracic echocardiography, tissue Doppler imaging, and aortic elasticity	FS	40 (5)	40 (4)	-	-
		EF	71 (4)	71 (5)	-	-
		RV MPI	0.42 (0.07)	0.43 (0.06)	0.42 (0.04)	0.43 (0.07)
		TV E/E′	6.87 (1.33)	6.84 (1.18)	6.75 (1.39)	6.98 (1.29)
		LV MPI	0.43 (0.05)	0.41 (0.05)	0.43 (0.06)	0.43 (0.05)
		MV E/E′	5.18 (0.92)	4.77 (0.94)	4.98 (1)	5.36 (0.83)
Amrousy **[29]** 2023 Egypt	Conventional transthoracic echocardiography, tissue Doppler imaging, and two-dimensional speckle-tracking echocardiography	FS	32.8 (2.4)	35.6 (6.5)	-	-
		EF	69 (8.1)	72.3 (6.9)	-	-
		RV MPI	-	-	-	-
		TV E/E′	-	-	-	-
		LV MPI	0.5 (0.1)	0.4 (0.09)	-	-
		MV E/E′	-	-	-	-
Biricini **[30]**		FS	38.2 (4.7)	41.4 (5.2)	-	-

2023<br>Turkey	ECG and conventional transthoracic echocardiography	EF	69.2 (5.3)	72.7 (5.8)	-	-
		RV MPI	-	-	-	-
		TV E/E′	-	-	-	-
		LV MPI	-	-	-	-
		MV E/E′	-	-	-	-
